# Impact of Persistent Anemia on Systemic Inflammation and Tuberculosis Outcomes in Persons Living With HIV

**DOI:** 10.3389/fimmu.2020.588405

**Published:** 2020-09-24

**Authors:** Fernanda O. Demitto, Mariana Araújo-Pereira, Carolina A. Schmaltz, Flávia M. Sant’Anna, María B. Arriaga, Bruno B. Andrade, Valeria C. Rolla

**Affiliations:** ^1^Programa de Pós-Graduação em Pesquisa Clínica em Doenças Infecciosas, Instituto Nacional de Infectologia Evandro Chagas, Fundação Oswaldo Cruz, Rio de Janeiro, Brazil; ^2^Instituto Gonçalo Moniz, Fundação Oswaldo Cruz, Salvador, Brazil; ^3^Faculdade de Medicina, Universidade Federal da Bahia, Salvador, Brazil; ^4^Multinational Organization Network Sponsoring Translational and Epidemiological Research (MONSTER) Initiative, Salvador, Brazil; ^5^Laboratório de Pesquisa Clínica em Micobacterioses (LAPCLIN-TB), Instituto Nacional de Infectologia Evandro Chagas, Fundação Oswaldo Cruz, Rio de Janeiro, Brazil; ^6^Escola Bahiana de Medicina e Saúde Pública (EBMSP), Salvador, Brazil; ^7^Universidade Salvador (UNIFACS), Laureate International Universities, Salvador, Brazil; ^8^Wellcome Centre for Infectious Diseases Research in Africa, Institute of Infectious Disease and Molecular Medicine, University of Cape Town, Cape Town, South Africa; ^9^Division of Infectious Diseases, Department of Medicine, Vanderbilt University School of Medicine, Nashville, TN, United States

**Keywords:** HIV, tuberculosis, anemia, inflammation, treatment outcome

## Abstract

Tuberculosis (TB) is associated with systemic inflammation and anemia, which are aggravated in persons living with HIV (PLWH). Here, we characterized the dynamics of hemoglobin levels in PLWH coinfected with TB undergoing antitubercular therapy (ATT). We also examined the relationships between anemia and systemic inflammatory disturbance as well as the association between persistent anemia and unfavorable clinical outcomes. Data on several blood biochemical parameters and on blood cell counts were retrospectively analyzed in a cohort of 256 TB/HIV patients from Brazil during 180 days of ATT. Multidimensional statistical analyses were employed to profile systemic inflammation of patients stratified by anemia status (hemoglobin levels <12 g/dL for female and <13.5 g/dL for male individuals) prior to treatment and to perform prediction of unfavorable outcomes, such as treatment failure, loss to follow up and death. We found that 101 (63.63%) of patients with anemia at pre-ATT persisted with such condition until day 180. Such individuals exhibited heightened degree of inflammatory perturbation (DIP), which in turn was inversely correlated with hemoglobin levels. Recovery from anemia was associated with increased pre-ATT albumin levels whereas persistent anemia was related to higher total protein levels in serum. Multivariable regression analysis revealed that lower baseline hemoglobin levels was the major determinant of the unfavorable outcomes. Our findings demonstrate that persistent anemia in PLWH during the course of ATT is closely related with chronic inflammatory perturbation. Early intervention to promote recovery from anemia may improve ATT outcomes.

## Introduction

Tuberculosis (TB) remains as a leading cause of death from infection by a single pathogen and also among people living with human immunodeficiency virus (HIV) (1). Persons living with HIV (PLWH) exhibit up to 19 times higher risk of developing active TB (2). In addition, TB is one of the most common opportunistic infections in PLWH. In fact, a total of 1.5 million people died from TB in 2018, including 251,000 PLWH (1). Understanding the determinants of clinical outcomes of PLWH coinfected with TB is critical to improve patient care.

Anemia is also a global public health problem and is diagnosed based on concentration of hemoglobin (Hb), specifically when it falls below established cut-off values; 12.0 g/dL for women and 13.5 g/dL for men (3). Low concentrations of Hb are a frequent complication of both TB and HIV infections, and its occurrence is associated with increased morbidity and mortality (4). Several causes of anemia are described, including iron deficiency and chronic inflammation (5–7). Prevalence of anemia in TB patients is reported to range between 32 and 96% (8), whereas in PLWH, this estimate varies from 1.3 to 95% (4). The extreme discrepancies in frequency of anemia associated with either TB and/or HIV infections published by several studies are thought to be influenced by factors that include study design, geographic location as well as clinical and epidemiological characteristics of patients.

Many studies have associated anemia with poor prognosis and increased mortality after TB diagnosis (6, 7, 9). In patients with TB, anemia has been attributed to be caused by chronic inflammation (10). It has also been shown that anemia is related to accelerated HIV/AIDS disease progression in PLWH (11). This latter study concluded that Hb levels is a robust biomarker to predict death independent of CD4^+^ T-cell count and HIV viral load values (11). More recently, a prospective investigation of antiretroviral therapy (HAART)-naïve PLWH reported that concurrent anemia and systemic inflammation were associated with higher risk of HAART failure (12). A potential explanation for the association between anemia and poor outcomes in HIV/AIDS and/or TB is that low Hb concentrations reflect more advanced disease staging. It is still to be defined the relationship between anemia and systemic inflammation in the context of antitubercular treatment (ATT) in PLWH and whether recovery from anemia during ATT in PLWH is related to improved prognosis.

In a study from Brazil, we have recently described that risk factors for mortality were distinct between HAART-naïve and HAART-experienced PLWH patients coinfected with TB. Indeed, in HAART-naïve patients, but not in those who were already undertaking antiretrovirals, the odds of death were substantially higher in patients who developed immune reconstitution inflammatory syndrome (IRIS) during the study follow up (13). This finding suggests that inflammation during the course of ATT in PLWH is related to unfavorable outcomes. In the present study, we expanded our analyses to investigate the relationship between the presence and severity of anemia and the cellular and biochemical profile of systemic inflammation in PLWH and TB in Brazil. We also tested whether low levels of Hb measured at pre-ATT could be used to predict unfavorable outcomes.

## Materials and Methods

### Ethics Statement

The study was approved by the Institutional Review Board of the Instituto Nacional de Infectologia Evandro Chagas (INI) (CAAE: 71191417.8.0000.5262). Written informed consent was obtained from all participants, and all clinical investigations were conducted according to the principles expressed in the Declaration of Helsinki.

### Population and Design

A prospective cohort has been followed at the Clinical Research Laboratory on Mycobacteria (LAPCLIN-TB) of the INI Evandro Chagas, Fundação Oswaldo Cruz, Rio de Janeiro, Brazil, since 2000. The present study is a retrospective assessment performed between 2008 and 2016, with data obtained from this cohort. Data were collected from electronic medical records based on standardized information of a defined template used in each patient’s visit for the whole cohort. PLWH 18 years and older, with clinical signs and symptoms of TB were included. The diagnosis of TB was made when *Mycobacterium tuberculosis* (Mtb) detection was positive in any sample collected (acid fast bacilli smear, Gene Xpert or culture from clinical specimens). In cases without bacteriological confirmation, the diagnosis was established by suggestive imaging analysis, histopathological examination, together with clinical and epidemiological findings consistent with TB. For those who had a negative culture, a positive therapeutic test with TB drugs was considered, after excluding other opportunistic diseases for differential diagnosis. Patients that initiated TB treatment and were diagnosed later with non-tuberculous mycobacteria as well as those who showed rifampicin and isoniazid resistance (multidrug resistance) were excluded. Patients with bone, mammary, renal or ocular TB were excluded, since these clinical forms can have very subtle, asymptomatic presentations, making it difficult to be compared to the other forms.

### Definitions

Anemia was defined according to World Health Organization (WHO) guideline criteria: Hb value < 13.5 g/dL for men and <12 g/dL for women.

Tuberculosis was classified as pleuropulmonary (when restricted to the lungs and/or pleura), extra-pulmonary (when just one extra-pulmonary site was identified) or disseminated (involving spleen, liver, bone marrow, or at least 2 non-contiguous sites).

Discharge due to cure, with or without etiologic confirmation of the diagnosis of TB, was considered a favorable outcome. Patients were defined as cured through clinical and/or radiologic improvement. Unfavorable outcome was defined as death, loss to follow up and treatment failure following the WHO guidelines. The cause of death was determined after thorough review of relevant clinical, microbiological and pathological data of each deceased patient.

### Antiretroviral and Antitubercular Therapies

Highly active antiretroviral therapy was offered according to contemporary Brazilian National Guidelines that were periodically updated (14). The first line ATT regimen was the combination of rifampicin, isoniazid and pyrazinamide during the two initial months, followed by rifampicin and isoniazid for 4 months, except when the continuation phase needed to be extended to 7 months such as in cases with central nervous system TB. From July 2009 on, ethambutol was added to the intensive phase regimen following a new recommendation of the National TB program of the Brazilian Ministry of Health (15). TB treatment scheme was adjusted in cases of severe adverse reactions, drug resistance and HAART regimens that precluded the use of rifampicin.

### Follow Up Visits

Visits included in this study were done at baseline, 60 and 180 days after TB therapy initiation. HAART were initiated after TB treatment according to decision from each physician and following the Brazilian TB treatment Guidelines (14). Information collected at the baseline visit included socio demographic data as well as previous TB and HAART, clinical presentation of TB, comorbidities like diabetes, hypertension, hepatitis (B and C), opportunistic diseases as well as CD4^+^ T-cell count and HIV VL among other variables. At baseline and in the follow up timepoints, patients underwent blood tests according to the INI’s clinical laboratory routine, with complete blood count and biochemical tests (creatinine, urea, total and direct bilirubin, albumin, alkaline phosphatase, uric acid, AST, GGT, ALT and total proteins).

Some patients (*n* = 06) who abandoned TB treatment (ATT loss to follow up) had recorded data on Complete Blood Count (CBC) and biochemical assessments in blood after the date of the outcome established by the present study (non-compliance), because those patients had been following up at INI by other specialties outside the TB outpatient clinic.

### Statistical Data Analysis

Three timepoints were considered: baseline, day 60 (D60) and day 180 (D180) of ATT. To perform baseline analysis, were used data from 256 patients. Due to lack of data in the subsequent timepoints (6.6% were missing data at D60 and 25.4% at D180), only 191 (74.6%) patients with complete laboratory data at all timepoints were considered for longitudinal analysis. Descriptive statistics was used to present data, use the median values with interquartile ranges (IQR) as measures of central tendency and dispersion, respectively, for continuous variables. Categorical variables were described using frequency (no.) and proportions (%). The Pearson chi-square test was used to compare categorical variables between study groups. The Mann–Whitney *U* test (for two unmatched groups), the Wilcoxon matched pairs test (for two matched groups), the Kruskal–Wallis test (for more than 2 unmatched groups) or the Jonckheere-Terpstra permutation and asymptotic test (for time series) were used to compare continuous variables. The Spearman rank test was used to assess correlations between indicated markers, conditions and timepoints. A multivariable logistic regression analysis model was used to identify independent determinants of persistent anemia and unfavorable treatment outcomes. The results were presented in the form of adjusted odds ratio (aOR) and 95% confidence intervals (CI).

The degree of inflammatory perturbation (DIP) is based molecular degree of perturbation (MDP) (16), an adaptation of the molecular distance to health previously described (17). In the present study, instead of using gene expression values, we inputted biochemical markers concentrations, HIV viral load and blood cells counts. Thus, herein, the average level and standard deviation of a baseline reference group (non-anemic at baseline) were calculated for each biomarker. The DIP score of each individual biomarker was defined by z-score normalization, where the differences in concentration levels from the average of the biomarker in reference group was divided by the reference standard deviation. The DIP score represents the differences by number of standard deviations from the control group.

Hierarchical cluster analysis (Ward’s method) using values of z-score normalized data was employed to depict the overall expression profile of indicated markers in the study subgroups. In this analysis, the dendrograms represent the Euclidean distance (inferring degree of similarity).

All analyses were pre-specified. Differences with *p*-values below 0.05 after adjustment for multiple comparisons (Holm-Bonferroni) were considered statistically significant. The statistical analyses. were performed using mdp (version 1.8.0), rstatix (version 0.4.0), stats (version 3.6.2), and caret (version. 6.0.86) R packages.

## Results

### Characteristics of the Study Participants

During the period from 2008 to 2016, 273 patients were screened, but 17 were excluded from all the analyses because of lack of data at baseline. Thus, the initial analysis included 256 patients, out of whom 219 (85.6%) were anemic and 37 (14.4%) were not anemic at baseline. The vast majority of study participants were male (71%), and the median age was 37 years old (IQR: 31–46). Individuals with anemia at baseline were similar to non-anemic participants with regard to, age, sex, overall frequency of comorbidities and life-habits (Table 1). Anemic patients more frequently self-reported weight loss (>10% of body weight) before initiating treatment and displayed lower CD4^+^ T-cell counts and higher HIV viral loads than those non-anemic at the study baseline (Table 1). Frequency of HAART use before TB diagnosis was higher in non-anemic study participants (65% in non-anemic vs. 43% in anemic, *p* = 0.021; Table 1).

**TABLE 1:** Characteristics of the study population.

**Characteristic**	**All (*n* = 256)**	**Anemic at baseline (*n* = 219)**	**Non-anemic at baseline (*n* = 37)**	***p*-value**
Age (years), median (IQR)	37 (31–46)	37 (30.7–46)	37 (33–46)	0.504
Sex, no. (% male)	182 (71)	157 (71.7)	25 (67.6)	0.996
Weight loss (>10%), no. (%)	190 (74.2)	174 (79.5)	16 (43.2)	<0.01
Smoking, no. (%)	131 (51.2)	114 (52)	17 (45.9)	0.948
Use of illicit drugs, no. (%)	74 (28.9)	60 (27.3)	14 (37.8)	0.185
Alcohol abuse^1^, no. (%)	88 (34.3)	80 (36.5)	8 (21.6)	0.133
Baseline CD4 count (cells/mm^3^), median (IQR)	170.5 (52–321.2)	153 (42.5–304.5)	294 (158–560)	<0.01
Baseline Viral Load log10 (copies/mL), median (IQR) (*n* = 165)	4.3 (1.69–0.5.23)	4.41 (2.5–5.31)	1.79 (1.69–4.22)	<0.01
D180 CD4 count (cells/mm^3^), median (IQR)	292.5 (165–432)	258 (157.5–403)	423 (266–603.5)	0.018
D180 Detectable Viral Load log10 (copies/mL), median (IQR) (*n* = 76)	3.21 (1.80–4.67)	3.17 (1.79–4.68)	3.42 (2.03–4.03)	0.766
D180 Undetectable VL, no. (%)	133 (52)	105 (65.2)	25 (38.5)	0.320
Days until outcome^2^, median (IQR)	189 (178.7–259.7)	189 (180–265)	189 (168–247.5)	0.564
Viral Hepatitis (B and/or C), no. (%)	25 (9.7)	21 (9.48)	4 (10.8)	0.945
Hypertension, no. (%)	21 (8.2)	16 (7.3)	5 (13.5)	0.270
Diabetes, no. (%)	32 (12.5)	28 (12.7)	4 (10.8)	0.946
Previous tuberculosis (%)	64 (25)	52 (23.7)	12 (32.4)	0.355
Complete TB treatment previous, no. (% of previous TB)	44 (68.8)	35 (67.3)	9 (75)	0.742
HAART use before TB, no. (%)	118 (46)	94 (42.9)	24 (64.8)	0.021
HAART during TB treatment, no. (%)	235 (91.7)	202 (92.3)	33 (89.2)	0.763
IRIS upon HAART initiation, no. (%)	12 (4.68)	12 (5.47)	0 (0)	–

*To define anemia according to baseline (D0) hemoglobin, the cut-off point of 12 g/dL for women and 13.5 g/dL for men was used. Data are shown as median and interquartile (IQR) range or frequency (percentage). Data were compared between the clinical groups using the Mann–Whitney *U* test (continuous variables) or the Pearson’s χ 2 test (for data on frequency). Complete data at baseline: 256 patients; Complete data at day 60: 239 (93.4%) patients, Complete data at day 180: 191 (74.6%) patients. ^1^The physicians also collected information about current use of illicit drugs and alcohol (Y/N to each) during the baseline interview. Potential problematic alcohol use was assessed with the CAGE questionnaire, with scores of 2 or greater indicating clinically significant alcohol problems. ^2^Outcomes: Favorable (cure) and Unfavorable (failure, loss follow-up or death). IQR, Interquartile Range; IRIS, Immune reconstitution Inflammatory Syndrome; TB, Tuberculosis; HAART, Highly Active Antiretroviral Therapy.*

To perform the longitudinal analysis, 82 of these patients were excluded because due to lack of data at some time point of the TB treatment (as described in “Materials and Methods”). Thus, 191 patients were further considered, out of whom 161 (84.3%) were anemic and 30 (15.7%) were not anemic at baseline. The median TB treatment period was 189 days for both groups. At day 180 of treatment, CD4^+^ T-cell counts increased in both study groups, but values in the group of participants who were anemic at the study baseline persisted substantially lower than those measured in non-anemic patients (*p* = 0.018; Table 1). Nevertheless, both frequency of individuals with undetectable HIV viral loads and median values with detectable viral loads were indistinguishable between study participants stratified based on anemia at baseline. There was no difference in the type of antitubercular treatment regimen between the study groups.

### Presence of Anemia Is Associated With Specific Cellular and Biochemical Profiles in Peripheral Blood of PWH Coinfected With TB

**FIGURE 1:**
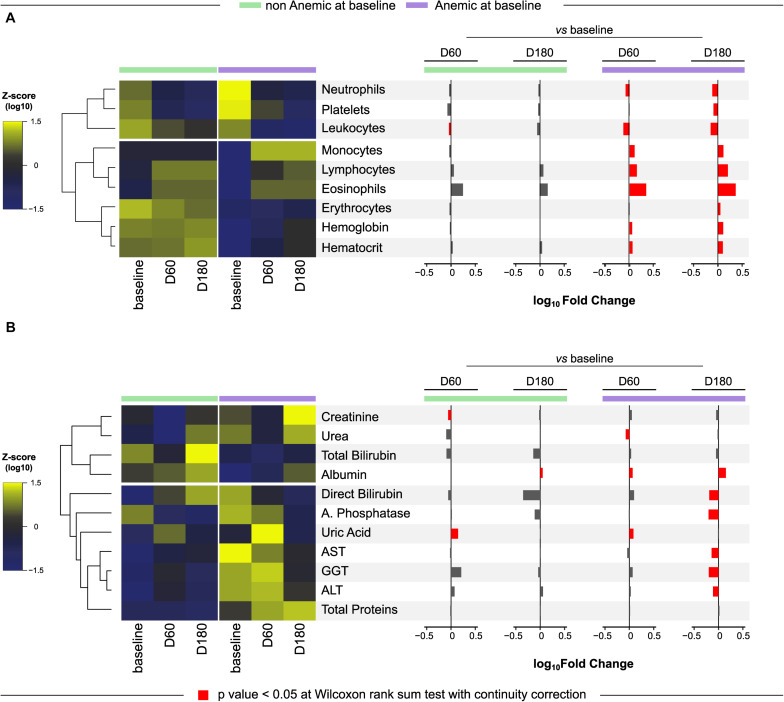
Differential change in biomarkers between anemic and non-anemic patients. A Heatmap was designed to depict the overall pattern of complete blood counts (CBC) **(A)** and biochemical markers **(B)** at all timepoints in anemic and non-anemic at different study timepoints of anti-tubercular treatment. A two-way hierarchical cluster analysis (Ward’s method) was performed. Dendrograms represent Euclidean distance. Expression scale represents Z-score normalization from the median at each timepoint and group. To define anemia according to baseline hemoglobin, the cut-off point of 12 g/dL for women and 13.5 g/dL for men was used. A log_10_ of fold-change was calculated and statistical analyses were performed using the Mann–Whitney *U* adjusted test. Significative differences (*p* < 0.05) between anemic and non-anemic patients for each time point are highlighted in red bars. Data are from 191 patients who had complete information on cell counts and biochemical measurements at all study timepoints.

The overall differences in cell counts and values of biochemical parameters measured at pre-ATT for anemic and non-anemic TB patients are described in Supplementary Table 1. As expected, erythrocyte counts, and values of hematocrit and hemoglobin were lower in anemic compared to non-anemic study participants. In addition, anemic patients exhibited lower counts of several leukocytes including lymphocytes and eosinophils at the study baseline (Supplementary Table 1). Additional analyses of the CBC parameters using hierarchical clustering of z-score normalized data and computation of fold change were performed to evaluate the dynamicity of the values over time in each group (Figure 1A). We observed a distinct profile between the groups, with three clusters defined in the heatmap, where the latter cluster (hemoglobin, hematocrit and erythrocyte) was the most consistent in both groups, with few changes mainly in the group of patients without anemia before treatment (baseline). Furthermore, it was possible to observe that, in the anemic participants, there was a significant difference in all parameters over time, mainly when comparing the baseline with the end of TB treatment (D180).

In regard to biochemical parameters, statistically significant differences were found in levels of ALT, AST and GGT, which were all higher in anemic patients at baseline, whereas the levels of albumin were lower (Supplementary Table 1 and Figure 1B). Additional hierarchical cluster and fold change analysis performed with biochemical parameters revealed a distinct profile between the groups (Figure 1B). Again, small changes over time in the group without anemia at baseline were observed, with increased levels of uric acid and decreased levels of creatinine at D60 and with increase in albumin levels at D180, comparing with baseline. In the group that presented anemia at baseline, the differences in levels of biomarkers were more pronounced. We found that, at D60, a decrease in urea levels and increase in uric acid and albumin levels were detected compared to baseline. At D180, there were significantly higher values of albumin and lower values of direct bilirubin, alkaline phosphatase, AST, GGT and ALT, than those measured at the study baseline.

### Correlation Between Cells and Biochemical Parameters With Hemoglobin

**FIGURE 2:**
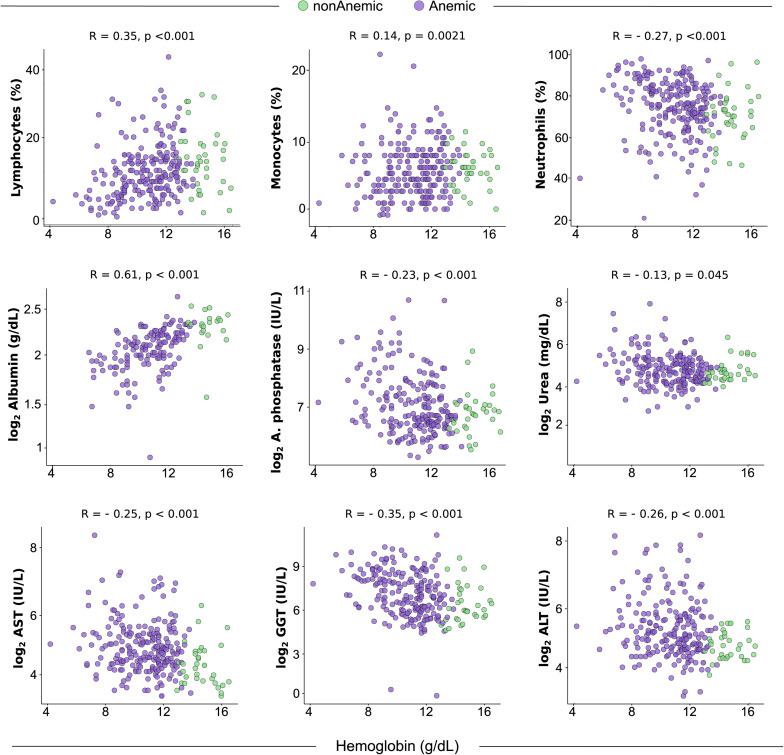
Spearman correlation analysis of cells and biochemical parameters versus hemoglobin in blood of TB-HIV prior to antitubercular treatment initiation. Plots from statistically significant Spearman correlations between biochemical parameters and hemoglobin levels at study baseline (pre-ATT) are shown (*n* = 256). To define anemia according to baseline hemoglobin, the cut-off point of 12 g/dL for women and 13.5 g/dL for men was used. ALT, Alanine Aminotransferase; AST, Aspartate Aminotransferase.

The results presented above indicate that anemia is associated with a distinct profile of cell counts and biochemical parameters in peripheral blood of patients with HIV-TB coinfection prior to initiation of ATT. We next examined the correlations between Hb levels and cell counts or values of the biochemical parameters (Figure 2). We observed that gradual increases in Hb values were related with decreases in percentage of neutrophils (*r* = −0.27; *p* < 0.001) and levels of ALT (*r* = −0.26; *p* < 0.001), AST (*r* = −0.25; *p* < 0.001), GGT (*r* = −0.35; *p* < 0.001), Alkaline Phosphatase (*r* = −0.23; *p* < 0.001), and Urea (*r* = −0.13; *p* = 0.045). Furthermore, frequency of lymphocytes (*r* = 0.35; *p* < 0.001) and monocytes (*r* = 0.14; *p* = 0.021), as well as levels of albumin (*r* = 0.61; *p* < 0.001) were increased proportionally to elevations in Hb levels (Figure 2). These findings reinforce the idea that degree of anemia is associated with changes in cellular and biochemical disturbances in peripheral blood.

### Dynamic Change of Hemoglobin Levels Upon Initiation of Anti-TB Treatment

**FIGURE 3:**
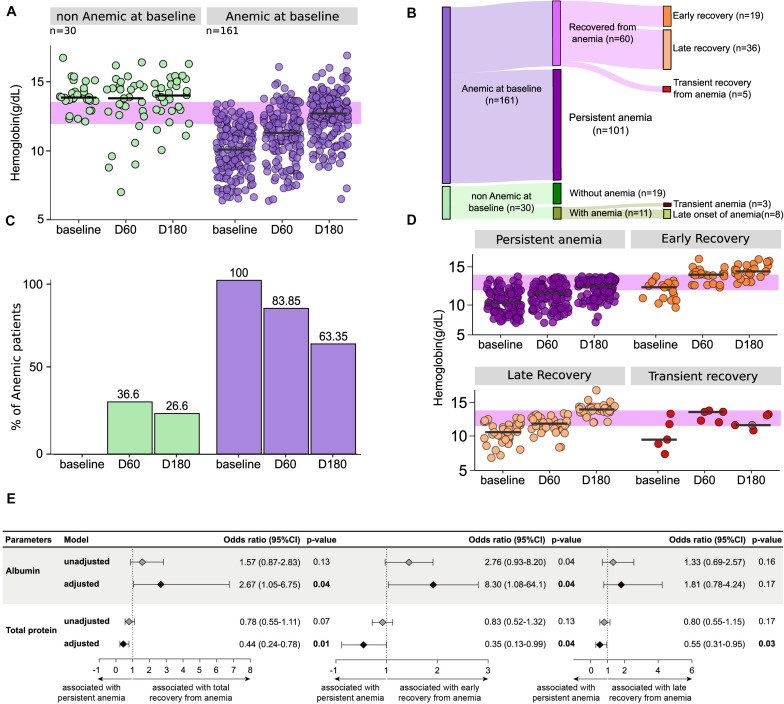
The majority of the anemic patients at baseline persist with low levels of hemoglobin after initiation of anti-tubercular treatment. To define anemia according to baseline hemoglobin, the cut-off point of 12 g/dL for women and 13.5 g/dL for men was used. **(A)** Hemoglobin levels at different time points of antitubercular therapy in the longitudinal population (*n* = 191) as well as in the groups of patients with or without anemia at baseline of treatment are shown. Anemic group presented statistically significant difference with *p* < 0.001 between baseline and timepoints after 2 months (D60 and D180) using Wilcoxon rank sum test with corrections. On Jonckheere-Terpstra permutation test, where an increase in one variable results in an increase or decrease in another variable, both groups presented *p* = 0.001 to “increase” hypothesis, with number of permutation equal to 1000. Using Jonckheere-Terpstra asymptotic test, *p*-value of non-anemic group was 0.851 and *p*-value of anemic group was <2.2e–16. Green dots represent non-anemic TB patients at baseline and purple dots represent anemic TB patients at baseline. **(B)** To define a patient as recovered from anemia, were considered normal levels (above the cut-off) of hemoglobin in any time point after D0. Chi-square test comparing D0 and D180 in both groups returned *p* < 0.00001. Green bars represent non-anemic TB patients at baseline and purple bars represent anemic TB patients at baseline. **(C)** Of the 161 patients who had anemia before starting treatment, 37.3% (*n* = 60) increased the values to normal hemoglobin levels at some time point. Of these, 95% were completely recovered (*n* = 55), so that 35% (*n* = 19) were recovered early (D60), and 60% (*n* = 36) were recovered late (D180). Finally, 5% (*n* = 5) of the patients who were anemic at study baseline presented a transient recovery (recovered at day 60 but were once again anemic at D180). 36.6% (*n* = 11) of the 30 patients without anemia at baseline developed anemia in D60, but three of them recovered normal hemoglobin values at D180. **(D)** Hemoglobin levels at different time points of antitubercular therapy in the in the population of anemic patients at baseline, divided according to the time of recovery. Using Jonckheere-Terpstra asymptotic test and Wilcoxon rank sum test with corrections, only the transient recovery group (that showed higher levels of hemoglobin at time 60 but had anemia at 180) did not exhibit a significant *p*-value between the timepoints. **(E)** Logistic binomial regression model was used to test independent associations between biochemical and clinical parameters and total recovery from anemia status at baseline, early recovery (recovery from anemia in ≤60 days from baseline) or late recovery (recovery from anemia in >60 days from baseline). The condition persistent anemia (anemia from baseline to day 180) was used as reference to test associations. Only parameters which remained with *p* ≤ 0.2 in univariate analysis (Supplementary Table 2 for details) model were inputted in the adjusted model. (95%CI, 95% confidence interval). Associations reported in **(E)** are for increases in 1 unit in plasma concentrations of the indicated markers. Data are from 191 patients who had complete information on cell counts and biochemical measurements at all study timepoints.

In order to better understand the impact of ATT commencement in the anemia, we prospectively investigated Hb levels at different time points of therapy (Figure 3). This approach revealed a differential dynamic of changes in Hb levels depending on the anemia status at the study baseline (Figure 3A). Indeed, a gradual increase in Hb levels over time on treatment was observed in the group of anemic participants (linear trend *p*-value: <0.001), whereas such levels did not substantially change in those who were not anemic at baseline. Curiously, 11 (36.6%) patients who were non-anemic at baseline developed anemia at day 60, from whom 8 (26.6% of the non-anemic group) were also anemic at day 180 of ATT (Figure 3B). Among the initially anemic patients, 83.85% were still anemic at day 60 and 63.35% persisted with anemia at day 180 of therapy (Figure 3B). A Sankey diagram was used to illustrate the dynamic change of anemia status over time on ATT (Figure 3C).

Hence, we observed that the vast majority of the participants who were anemic at the study baseline persisted with anemia until at least day 180 of therapy, whereas 19 (11.8%) individuals recovered from anemia at day 60 (early recovery), 36 (22.36%) recovered only by day 180 (late recovery), and 5 (3.1%) recovered at day 60 but were once again anemic at day 180 (transient recovery). The characteristics of these subpopulations are shown in the Supplementary Table 2. The dynamicity of hemoglobin levels in the different subgroups of anemic patients identified in the Sankey diagram is described in Figure 3D. Among the patients who had anemia at the baseline, with the exception of the transient recovery group, all exhibited a significant increase in hemoglobin levels over time of ATT (*p*-values < 0.05) (Figure 3D).

### Persistent Anemia Is Associated With Augmented Degree of Inflammatory Perturbation

**FIGURE 4:**
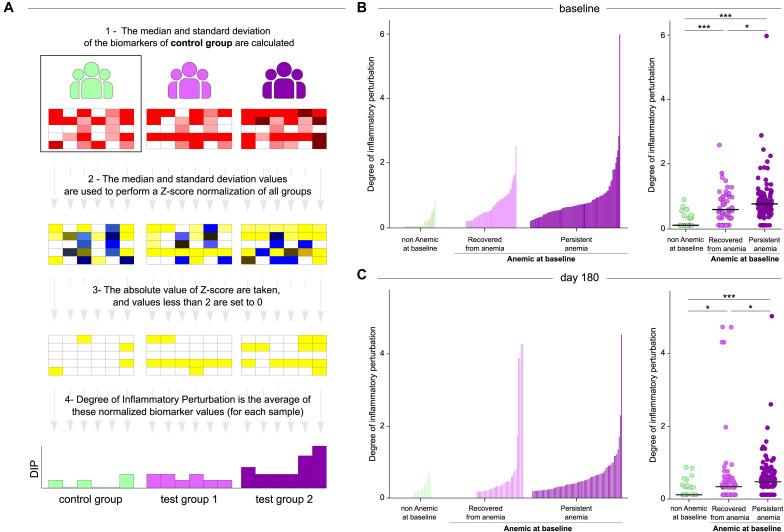
Patients who persists with anemia exhibit substantially higher degree of inflammatory perturbation. **(A)** Degree of inflammatory perturbation (DIP) is based on Molecular Degree of Perturbation (16), but instead of using gene expression, we use biochemical and cellular markers. DIP was calculated was calculated using the median and standard deviation of the control group as a starting point. Then, the Z-score was calculated for all groups, a cut-off point was established and, finally, an average disturbance calculation was performed for each sample. This figure is adapted https://mdp.sysbio.tools/about. **(B,C) Left panels:** Histograms show the single sample degree of inflammatory perturbation (DIP) score values relative to the non-anemic at baseline group between patients recovered from anemia and with persistent anemia at baseline **(B)** and day 180 **(C)**. **Right panels:** Scatter plots of the summary data for each group are shown. DIP score values were compared between non-anemic at baseline (control group), recovered from anemia and persistent anemia at baseline **(B)** and day 180 **(C)**. Lines in the scatter plots represent median values. Data were compared using the Mann–Whitney *U* test. *P < 0.05; ***P < 0.0001. Data are from 191 patients who had complete information on cell counts and biochemical measurements at all study timepoints.

Given that the majority of anemic patients persisted with anemia during the time of ATT regardless of the gradual increase in hemoglobin levels, we tested whether such condition was related to a chronic and unresolved inflammatory disturbance. To do so, we employed a mathematical maneuver named Molecular Degree of Perturbation (MDP), which has been used by our group and others to estimate the overall degree of inflammation and/or immune activation (18–20). In the present study, we included cells (from CBC), viral load, CD4 counts and biochemical parameters (creatinine, urea, total and direct bilirubin, albumin, alkaline phosphatase, uric acid, AST, GGT, ALT and total proteins) to create a score henceforth named Degree of Inflammatory Perturbation (DIP) (Figure 4A). We found that in general, anemia was associated with increased DIP values measured at both baseline (Figure 4B) and at day 180 of ATT (Figure 4C), with the highest levels being detected in the group of persistent anemia. Strikingly, the DIP score values exhibited strong inverse correlations with hemoglobin levels both at baseline (*r* = −0.74; *p* < 0.001) and at day 180 (*r* = −61; *p* < 0.001), highlighting that the degree of anemia and activation of inflammation are concurrent processes.

Additional analyses demonstrated that, as expected, patients who had an early recovery from anemia exhibited significantly higher baseline values for erythrocytes, Hb, hematocrit, neutrophils (Supplementary Figure 1) and albumin (Supplementary Figure 2) than those who did not recover. Patients who had a late recovery displayed significantly higher baseline values of Hb and hematocrit compared to those who persisted anemic (Supplementary Figures 1, 2). The prospective comparisons have also identified discrepancies in cell counts and concentrations of biochemical parameters between the subgroups of patients based on recovery from anemia, which are summarized in Supplementary Figures 1, 2.

The findings described above led us to hypothesize that the distinct profile of cell counts, and levels of biochemical parameters, measured at pre-ATT, is associated with persistent anemia. Thus, a stepwise binary multivariate logistic regression analysis was performed to test if biochemical parameters measured at pre-ATT (baseline) are able to predict recovery from anemia. Results demonstrated that increases in concentrations of albumin were directly associated with recovery from anemia (aOR: 2.67, 95% CI:1.05–6.75, *p* = 0.04) whereas increases in total proteins were directly associated with persistent anemia (aOR: 0.44, 95% CI: 0.24–0.78, *p* = 0.01) (Figure 3E). Similar trends in associations were observed when the major group of participants who recovered from anemia were further stratified in early and late recovery.

### Lower Concentrations of Hemoglobin at Pre-ATT Are Associated With Increased Risk of Unfavorable Treatment Outcome

**FIGURE 5:**
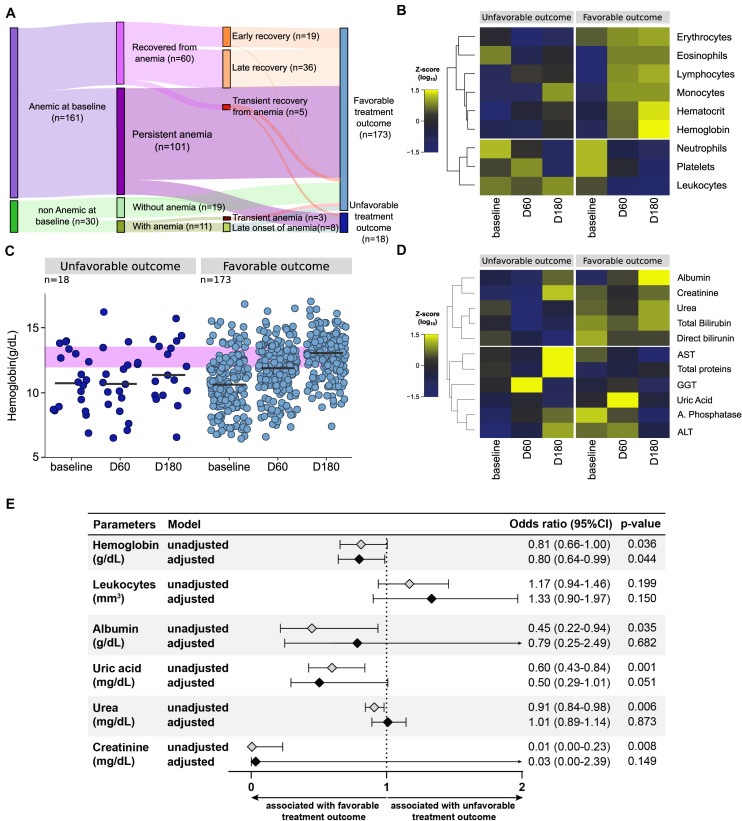
Unfavorable outcome occurs mainly in patients with anemia. **(A)** Among all 191 patients who had complete laboratory data from all the study timepoints, only 18 had unfavorable treatment outcomes (death, failure or loss to follow up), whereas 173 were successfully treated (cure). **(B)** 14 of the 18 patients had anemia at baseline and 14 also had anemia at the end of the treatment. **(C,D)** A Heatmap was designed to depict the overall pattern of cells and biochemical markers at all timepoints in favorable and unfavorable outcome of anti-tubercular treatment. A two-way hierarchical cluster analysis (Ward’s method) was performed. Dendrograms represent Euclidean distance. Expression scale represents Z-score normalization from the median at each timepoint and group. It is possible to observe that patients who had an unfavorable outcome have a profile opposite to that of patients with a favorable outcome. In **(C)** we can identify that patients with a favorable outcome had an increase in most of the blood count parameters over time, and also a decrease in neutrophils, platelets and leukocytes, returning to normal levels of these components. In patients with an unfavorable outcome, the parameters remain more similar to the baseline, with the exception of neutrophils and platelets, which also decrease. In **(D)** the biochemical markers of both groups do not seem to change much over time, but they visibly present different profiles when comparing favorable and unfavorable. Patients with a favorable outcome have higher levels of albumin, creatinine, urea and bilirubin throughout the treatment. **(E)** Logistic binomial regression model to significant biochemical parameters was used to test independent associations between biochemical and clinical parameters at baseline and treatment outcome after 180 days. The treatment outcome unfavorable (failure, death or loss to follow up) was used as reference to test associations. Only parameters which remained with *p* ≤ 0.2 in univariate analysis model were inputted in the adjusted model. (95%CI, 95% confidence interval). Associations reported in **(E)** are for increases in 1 unit in plasma concentrations of indicated markers.

In the longitudinal study cohort, 18 patients (9.4%) developed unfavorable outcomes (death attributed to TB: *n* = 3; death attributed to HIV: *n* = 2; ATT failure: *n* = 1; ATT loss to follow up abandonment: *n* = 12). The majority of the cases of unfavorable outcomes was composed by individuals who experienced persistent anemia (14 out of 18 participants, 77.8%) (Figure 5A). In fact, the median values of Hb levels gradually increased upon initiation of ATT in patients who were successfully treated (linear trend *p* < 0.001) but did substantially change in those who has unfavorable outcomes (Figure 5B). A hierarchical cluster analysis inputting average values of CBC (Figure 5C) and biochemical parameters (Figure 5D) demonstrated that there were differential trends in values between the study timepoints and the subgroups of favorable vs. unfavorable outcomes.

At study baseline, individuals who further developed unfavorable outcomes exhibited lower levels of Hb (*p* = 0.052), albumin (*p* = 0.035), uric acid (*p* = 0.001), urea (*p* = 0.006), and creatinine (*p* = 0.008) than those who were further successfully treated (Supplementary Table 3). A binomial logistic regression analysis was performed to test independent associations between the parameters analyzed and treatment outcome (Figure 5E). We found that increases in hemoglobin at pre-ATT were protective against unfavorable outcomes (aOR: 0.80, 95% CI: 0.64–0.99, *p* = 0.04) independent of the other factors (Figure 5E). These results highlight the importance of Hb as a prognostic marker in PLWH coinfected with TB.

## Discussion

Anemia is a common complication associated with both TB and HIV, and it has been reported to occur in between 16 and 94% of TB patients (21–24); whereas in PLWH the prevalence ranges from 39 to 71% (25–27). These observations were validated by the present study, which was focused on TB-HIV coinfection, and reported that 84.3% of the study participants were anemic at pre-ATT. In addition, our findings demonstrated that anemic patients exhibit higher inflammatory perturbation in the peripheral blood, which is sustained over the course of ATT in those who persisted with low Hb levels. Such condition is shown here to be closely associated with unfavorable outcomes. Early intervention focused on recovery from anemia could be a strategy to optimize the clinical management of PLWH with TB during ATT treatment.

In our cohort, anemic patients more frequently exhibited weight loss, lower CD4^+^ T-cell counts and higher HIV viral loads than those who were not anemic. These observations reinforce the idea that anemia infers more advanced stage of disease progression. Our results are in agreement with other previously published findings which demonstrated that lower body mass index (27–29), higher HIV viral loads (28), and lower CD4^+^ T-cell counts are all associated with higher prevalence of anemia (25, 26). As previously reported by us in a different cohort of TB patients, most of the anemia cases are attributed to chronic inflammation rather than to iron deficiency (10). A recent systematic review demonstrated that anemia is related to an increased risk of all-cause mortality and incident TB among PLWH, regardless of the anemia type (30). The magnitude of such effect is thought to be proportional to severity of anemia. Finally, iron supplementation in such cases is still a matter of debate, with inconsistent results reported by clinical trials. The probable determinants of anemia in the context of HIV/AIDS and TB are likely multifactorial and involve several factors including nutritional status (31), chronic inflammation and antibody-mediated erythrophagocytosis (32). Our results demonstrated that anemic patients also exhibit lower counts of other cell types, suggesting that a global effect on the bone marrow may be occurring. Additional mechanistic studies as well as large randomized clinical trials testing different approaches to reduce anemia are necessary to improve our knowledge regarding the molecular targets and to help delineate the best therapeutic schemes.

With regard to the biochemical parameters, our results indicate that low Hb levels accompanied higher values of ALT, AST and GGT, and lower concentrations of albumin. Such findings are similar to those previously published by our group in another cohort of TB patients and reinforce the idea that anemia is related to a distinct biochemical profile and linked to inflammation (10). In our study, the prevalence of hepatitis B or C in anemic patients (9.48%) was very similar to non-anemic patients (10.8%), suggesting that although this comorbidity is present, it is probably not the main factor driving the differences in the levels of liver transaminases. At the end of ATT, none of these biochemical markers demonstrated association with the clinical outcomes. Moreover, out of the 25 patients who had viral hepatitis, 20 (80%) had a favorable outcome, highlighting the low influence of this coinfection on the effectiveness of the treatment.

The results reported here demonstrated that among the study participants with anemia at the baseline, the vast majority persisted with low Hb levels until day 180 of ATT. In addition, within the group of patients who recovered from anemia under the course of ATT, most exhibited a late recovery, occurring between day 60 and day 180 of therapy. Other investigations have reported that anemia frequently has a benign course in TB patients without HIV coinfection, with complete recovery in 64.5% of patients undertaking ATT (5). The discrepancies between the findings presented here and this previous study can be likely explained but the fact that our cohort was composed by PLWH, which may have an additional detrimental effect on inflammation and its related anemia compared to the setting of TB in the absence of HIV. In our study, patients who recovered from anemia presented with relatively higher values of Hb and hematocrit at baseline compared to those who persisted anemic. Individuals who had early recovery from anemia also exhibited higher neutrophil counts and albumin levels. The multivariable logistic regression analysis performed here revealed that albumin was independently associated with recovery from anemia. This observation again reinforces the strong association of albumin levels with recovery from anemia. These findings suggest that the degree of anemia is associated with changes in concentrations of cells and biochemical markers and that more severe anemia before ATT indicates higher odds of persistent anemia for up to 6 months on therapy.

To describe the overall biochemical and cellular disturbances related to anemia in the study population, we used an adaption of the molecular degree of perturbation (18) to estimate the degree of inflammatory perturbation in PLWH and with TB according to anemia status. Our findings indicate that there are important discrepancies in the DIP values between patients with persistent anemia compared to those who recovered during ATT. Individuals who persisted with anemia in the course of ATT exhibited higher DIP values already at pre-ATT, and such profile was sustained ay day 180 of therapy. These findings argue that persistent anemia directly associates with increased disturbances in the biochemical and cellular profiles, which were sustained over the course of ATT. The inverse correlations between DIP values and Hb levels both at pre-ATT and at day 180 indicate that the degree of inflammatory perturbation is proportional to the severity of anemia. Whether anemia sets the stage for persistent inflammation or is just a hallmark of chronic, unfettered, dysregulation of inflammatory responses warrants further investigation. This association between low Hb levels and risk of inflammatory disturbance has been described in PLHW who experience IRIS (33, 34) and also in patients with HIV/TB coinfection (35).

Another important contribution of our study was to test whether lower concentrations of Hb at pre-ATT could be used to predict risk of unfavorable outcomes. We found that the majority of patients who had unfavorable outcomes experienced persistent anemia during the course of ATT. A previous study described that anemia is associated with a 2–3 times increase in the risk of death, recurrence of TB or ATT failure in PLWH/TB (7). Corroborating with these findings, the results from a logistic regression analysis presented here demonstrated that increases in Hb concentrations at pre-ATT play a protective role against unfavorable outcomes independent of other confounding factors.

Our study has some limitations, such as relatively small number of non-anemic participants and of unfavorable outcomes, although the latter is within the expected range in the outpatient clinic from our institution. The small sample size favors a potential bias, as well as the fact that we do not have data on these same patients prior to TB and/or HIV infection, so that we cannot determine whether the anemia was pre-existing or in fact is a consequence of the co-infection. The study population also included few IRIS cases, which precluded additional exploratory analyses. Regardless of such limitations, our study adds to the current knowledge in the field by demonstrating the relevance of persistent anemia in driving inflammatory disturbances related to worse prognosis of PLWH coinfected with TB. The fact that most patients with an unfavorable outcome persisted with anemia and with a high degree of inflammatory perturbation suggests that early intervention focused on recovery from anemia could be a strategy to optimize the clinical management of PLWH with TB during ATT treatment.

## Data Availability Statement

The raw data supporting the conclusions of this article will be made available by the authors, without undue reservation.

## Ethics Statement

The studies involving human participants were reviewed and approved by the Institutional Review Board of the Instituto Nacional de Infectologia Evandro Chagas (INI). The patients/participants provided their written informed consent to participate in this study.

## Author Contributions

FD, CS, FS’A, and VR contributed to conception and design of the study. FD also collected the data and organized the database. MA-P and MA performed the statistical analysis and data visualization. FD, MA-P, and BA wrote the first draft of the manuscript. VR and BA supervised the project execution. All authors contributed to the article and approved the submitted version.

## Conflict of Interest

The authors declare that the research was conducted in the absence of any commercial or financial relationships that could be construed as a potential conflict of interest.
