# Supplementary material for: Impact of Persistent Anemia on Systemic Inflammation and Tuberculosis Outcomes in Persons Living With HIV
Source: Front Immunol. 2020 Sep 24;11:588405. doi: 10.3389/fimmu.2020.588405 (PMC7541849; doi:10.3389/fimmu.2020.588405)
Supplement: Supplementary file 1 [file Table_1.DOCX]

Supplementary Material

**Supplementary Table 1. Concentrations of cells and biomarkers in peripheral blood, according to anemia status at study baseline (n=256).**

| **Count Blood Cells and Hemoglobin** | | | |  | **Biochemical markers** | | | |
| --- | --- | --- | --- | --- | --- | --- | --- | --- |
|  | **Median (IQR1- IQR3)** | |  |  |  | **Median (IQR1- IQR3)** | |  |
|  | **Anemic**  **(n= 219)** | **non-Anemic**  **(n= 37)** | **p-value** |  |  | **Anemic**  **(n= 219)** | **non-Anemic**  **(n= 37)** | **p-value** |
| Basophils (%) | 0(0-0) | 0(0-0) | - |  | Albumin (g/dL) | 2.6(2.1-3.1) | 3.6(3.3-3.8) | **< 0.01** |
| Eosinophils (%) | 1(0-2) | 2(1-4) | **0.014** |  | A. Phosphatase (IU/L) | 125(97.5-236) | 130(97-156) | 0.306 |
| Erythrocytes (million/mm^3^) | 3.6(3.1-4.1) | 4.6(4.1-4.8) | **< 0.01** |  | ALT (IU/L) | 39(28-58.3) | 30.4(24-38.5) | **< 0.01** |
| Hematocrit (%) | 30.4(26.8-34.5) | 39.8(35.8-42.4) | **< 0.01** |  | AST (IU/L) | 40.5(29-61) | 26(18.8-38.3) | **< 0.01** |
| Hemoglobin (g/dL) | 10.6(8.9-11.5) | 13.9(13.3-14.8) | **< 0.01** |  | Creatinine (mg/dL) | 0.87(0.72-1.1) | 0.83(0.74-0.94) | 0.42 |
| Leukocytes(mm^3^) | 6550(4400-8770) | 6630(5170-8570) | 0.41 |  | Dir. Bilirubin (mg/dL) | 0.15(0.1-0.26) | 0.12(0.07-0.21) | **0.09** |
| Lymphocytes (%) | 18(12-26) | 25(16-33) | **< 0.01** |  | GGT (U/L) | 132(64.5-323) | 64(44-173) | **< 0.01** |
| Monocytes (%) | 7(4-10) | 8(6-10) | 0.20 |  | Tot. bilirubin  (mg/dL) | 0.36(0.25-0.59) | 0.43(0.29-0.62) | 0.52 |
| Neutrophils (%) | 71(60.5-80) | 64(56-72) | **0.016** |  | Tot. proteins (mg/dL) | 8.2(7.7-9.1) | 8(7.5-8.6) | 0.44 |
| Platelets (mil/mm^3^) | 294(216-379) | 292(232-326) | 0.75 |  | Urea (mg/dL) | 25(17-34) | 22(18.5-25.6) | 0.54 |
|  |  |  |  |  | Uric acid (mg/dL) | 5.1(3.4-9.1) | 4.4(3.5-5.75) | 0.52 |

**Table note:**

Bold font indicates statistical significance.

^1^To define anemia according to baseline (D0) hemoglobin, the cut-off point of 12g/dL for women and 13.5g/dL for men was used. Abbreviations: IQR: Interquartile range; ALT: Alanine Aminotransferase; AST: Aspartate Aminotransferase; GGT: Gamma-Glutamyl Transferase;

Data are shown as median and interquartile (IQR) range or frequency (percentage). Data were compared between the clinical groups using the Mann-Whitney *U* test (continuous variables).

Clinical laboratory assessment was measured by cell blood count and specific biochemical tests as described in Methods.

**Supplementary Table 2. Characteristics of the study population and concentrations of cells and biomarkers in peripheral blood, according recovered from anemia.**

| **Characteristics** | **Total recovery**  **(n=55)** | **Early recovery**  **(n=19)** | **Late recovery (n=36)** | **Persistent anemia (n=99)** | **p-value**  **< 0.05^2^** |
| --- | --- | --- | --- | --- | --- |
| Sex. no. (% male) | 44 (78.6) | 15 (71.4) | 29 (82.9) | 70 (70.7) | ns |
| Age (years). no. (IQR) | 37 (28-44) | 34 (26-41) | 39 (29-46) | 38 (31-47) | ns |
| Smoking. no. (%) | 30 (53.6) | 10 (47.6) | 20 (57.1) | 47 (48) | ns |
| Use of illicit drugs. no. (%) | 11 (19.6) | 2 (9.5) | 9 (25.7) | 27 (27.3) | ns |
| Alcohol abuse^1^, no. (%) | 20 (35.7) | 7 (33.3) | 13 (37.1) | 37 (37.8) | ns |
| Weight loss (>10%) no. (%) | 47 (83.9) | 18 (85.7) | 29 (82.9) | 79 (79.8) | ns |
| Hypertension. no. (%) | 4 (7.4) | 1 (5) | 3 (8.8) | 9 (9.1) | ns |
| Diabetes. no. (%) | 5 (8.9) | 2 (9.5) | 3 (8.6) | 14 (14.1) | ns |
| Previous tuberculosis, no. (%) | 10 (17.9) | 5 (23.8) | 5 (14.3) | 19 (19.2) | ns |
| Complete TB treatment previous, no. (% of prior TB) | 7 (70) | 4 (80) | 3 (60) | 14 (73.7) | ns |
| HAART use before TB, no. (%) | 20 (35.7) | 8 (38.1) | 12 (34.3) | 31 (31.3) | ns |
| HAART during TB treatment, no. (%) | 55 (98.2) | 20 (95.2) | 35 (100) | 92 (92.9) | ns |
| IRIS upon HAART initiation, no. (%) | 3 (5.4) | 1 (4.8) | 2 (5.7) | 5 (5.1) | ns |
| **Count Blood Cells** |  |  |  |  |  |
| Erythrocytes (million/mm^3^) | 3.96 (3.48-4.23) | 4.05 (3.65-4.28) | 3.82 (3.48-4.22) | 3.57 (3.01-4.12) | **a, b, c** |
| Hemoglobin (g/dL) | 10.8 (9.6-11.95) | 11.8 (9.8-12.3) | 10.6 (9.4-11.7) | 9.4 (8.1-11.1) | **a, b, c** |
| Hematocrit (%) | 32.6 (28.9-35.9) | 34.1 (30.3-36.3) | 31.9 (28.9-35.4) | 28.9 (24.7-33.6) | **a, b, c** |
| Leukocytes(mm^3^) | 6.91 (4.65-8.89) | 7.22 (6.27-8.88) | 6.03 (4.64-9.53) | 6.1 (4.23-8.52) | **b** |
| Eosinophils (%) | 0 (0-2) | 0 (0-1) | 1 (0-3) | 1 (0-2) | **b** |
| Basophils (%) | 0 (0-0) | 0 (0-0) | 0 (0-0) | 0 (0-0) | ns |
| Neutrophils (%) | 74.5 (61.5-81) | 76 (73-80) | 64 (58-82) | 70 (61-80) | **b** |
| Lymphocytes (%) | 17.5 (12-26.5) | 15 (12-18) | 24 (11-27) | 18 (12-26) | ns |
| Monocytes (%) | 8 (3.5-11) | 7 (4-8) | 8 (3-12) | 6 (4-10) | ns |
| Platelets (mil/mm^3^) | 3.01 (2.42-3.69) | 3.07 (2.4-3.54) | 3.01 (2.47-4.02) | 2.9 (2.08-3.78) | ns |
| **Biochemical markers** |  |  |  |  |  |
| Tot. proteins (mg/dL) | 8.1 (7.4-8.7) | 7.9 (7.35-8.25) | 8.1 (7.4-8.8) | 8.4 (7.6-9.25) | **a** |
| Albumin (g/dL) | 2.8 (2.2-3.2) | 3.1 (2.65-3.2) | 2.69 (2.03-3.2) | 2.6 (2-3) | **b** |
| ALT (IU/L) | 41 (29-65) | 44 (27.5-65.5) | 40 (29-65) | 40 (30.5-61.5) | ns |
| AST (IU/L) | 42 (32-63) | 49.25 (38.5-62.09) | 39.3 (31-78) | 39 (29.32-61) | ns |
| A.Phosphatase (IU/L) | 132 (91-256.34) | 171.26 (87-242.64) | 130 (98.3-315) | 148 (96.5-244) | ns |
| GGT (U/L) | 117 (70-377) | 114 (70-253) | 117 (87-420) | 170 (74-369) | ns |
| Tot. bilirubin (mg/dL) | 0.39 (0.31-0.58) | 0.45 (0.27-0.58) | 0.4 (0.3-0.6) | 0.37 (0.25-0.6) | ns |
| Dir. Bilirubin (mg/dL) | 0.16 (0.11-0.27) | 0.19 (0.11-0.26) | 0.2 (0.1-0.3) | 0.18 (0.12-0.29) | ns |
| Uric acid (mg/dL) | 4.2 (3.7-8.3) | 4.2 (3.7-5.8) | 4.4 (3.7-9.2) | 5.7 (3.6-9.6) | ns |
| Urea (mg/dL) | 24.07 (18.09-30.88) | 20 (17-29) | 25 (20-33) | 27 (18-35) | ns |
| Creatinine (mg/dL) | 0.86 (0.74-1.11) | 0.86 (0.78-1.06) | 0.9 (0.7-1.2) | 0.85 (0.71-1.09) | ns |

**Table note:**

Bold font indicates statistical significance. To define anemia according to baseline (D0) hemoglobin, the cut-off point of 12 g/dL for women and 13.5 g/dL for men was used. Data are shown as median and interquartile (IQR) range or frequency (percentage). Data were compared between the clinical groups using the Mann-Whitney *U* test (continuous variables) or the Pearson’s χ 2 test (for data on frequency).

^1^The physicians also collected information about current use of illicit drugs and alcohol (Y/ N to each) during the baseline interview. Potential problematic alcohol use was assessed with the CAGE questionnaire, with scores of 2 or greater indicating clinically significant alcohol problems.

^2^  Significance: ns: not significant in all comparisons; a (significant in total recovery versus persistent anemia), b (significant in early recovery versus persistent anemia), c (significant in late recovery versus persistent anemia).

Abbreviations: IQR: Interquartile Range; IRIS: Immune reconstitution Inflammatory Syndrome; TB: Tuberculosis; HAART: Highly Active Antiretroviral Therapy;

**Supplementary Table 3. Characteristics of the study population and concentrations of cells and biomarkers in peripheral blood, according to treatment outcome.**

| **Characteristics** | **Unfavorable treatment outcome (n=18)** | **Favorable treatment outcome (n=172)** | **p-value** |
| --- | --- | --- | --- |
|  |  |  |  |
| Sex. no. (% male) | 10 (55.6) | 129 (75.0) | 0.094 |
| Age (years). no. (IQR) | 37 (26-44) | 38 (31-46) | 0.546 |
| Smoking. no. (%) | 9 (50.0) | 83 (49.4) | 1.000 |
| Use of illicit drugs. no. (%) | 7 (38.9) | 41 (24.1) | 0.253 |
| Alcohol abuse^1^, no. (%) | 6 (33.3) | 57 (33.5) | 1.000 |
| Weight loss (>10%) no. (%) | 13 (72.2) | 127 (74.3) | 0.785 |
| Hypertension. no. (%) | 0 (0.0) | 16 (9.5) | 0.369 |
| Diabetes. no. (%) | 1 (5.6) | 21 (12.2) | 0.700 |
| Previous tuberculosis, no. (%) | 4 (22.2) | 35 (20.3) | 0.776 |
| Complete TB treatment previous, no. (% of prior TB) | 1 (25.0) | 29 (82.9) | 0.032 |
| HAART use before TB, no. (%) | 8 (44.4) | 63 (36.6) | 0.610 |
| HAART during TB treatment, no. (%) | 13 (72.2) | 164 (95.3) | **0.003** |
| IRIS upon HAART initiation, no. (%) | 1 (5.6) | 8 (4.7) | 0.600 |
| **Count Blood Cells** |  |  |  |
| Erythrocytes (million/mm^3^) | 3.74 (3.17-4.32) | 3.87 (3.16-4.32) | 0.898 |
| Hemoglobin (g/dL) | 10.6 (8.9-12.15) | 10.8 (8.6-12.9) | **0.052** |
| Hematocrit (%) | 28.75 (26-36.2) | 32.1 (27.1-35.9) | 0.316 |
| Leukocytes(mm^3^) | 6.46 (4.31-8.27) | 6.56 (4.65-8.8) | 0.199 |
| Eosinophils (%) | 3 (1-6) | 1 (0-2) | **0.013** |
| Basophils (%) | 0 (0-0) | 0 (0-0) | 0.573 |
| Neutrophils (%) | 69 (65-74) | 70 (60-80) | 0.986 |
| Lymphocytes (%) | 21 (10-28) | 19 (12-27) | 0.914 |
| Monocytes (%) | 7 (5-9) | 7 (4-10) | 0.883 |
| Platelets (mil/mm^3^) | 3 (2-4) | 3 (2-4) | 0.901 |
| **Biochemical markers** |  |  |  |
| Tot. proteins (mg/dL) | 8 (6-9) | 8 (8-9) | 0.521 |
| Albumin (g/dL) | 3 (2-4) | 3 (2-3) | **0.035** |
| ALT (IU/L) | 35 (30-61) | 39 (28-56) | 0.697 |
| AST (IU/L) | 39 (31-55) | 39 (28-60) | 0.908 |
| A.Phosphatase (IU/L) | 121 (90-173) | 135 (97-240) | 0.387 |
| GGT (U/L) | 161 (81-363) | 132 (63-341) | 0.536 |
| Tot. bilirubin (mg/dL) | 0 (0-1) | 0 (0-1) | 0.275 |
| Dir. Bilirubin (mg/dL) | 0 (0-0) | 0 (0-0) | 0.384 |
| Uric acid (mg/dL) | 5 (4-9) | 5 (3-8) | **0.001** |
| Urea (mg/dL) | 24 (18-26) | 25 (18-35) | **0.006** |
| Creatinine (mg/dL) | 1 (1-1) | 1 (1-1) | **0.008** |

**Table note:**

Favorable outcome: Cure; Unfavorable outcome: Death, loss to follow up and treatment failure

Abbreviations: IQR: Interquartile Range; TB: Tuberculosis; HAART: Highly Active Antiretroviral Therapy; ALT: Alanine Aminotransferase; AST: Aspartate Aminotransferase; GGT: Gamma-Glutamyl Transferase; IRIS: Immune Reconstitution Inflammatory Syndrome Data are shown as median and interquartile (IQR) range or frequency (percentage). Data were compared between the clinical groups using the Mann-Whitney *U* test (continuous variables) or the Pearson’s χ 2 test (for data on frequency). Data are from 191 patients who had complete information on cell counts and biochemical measurements at all study timepoints.


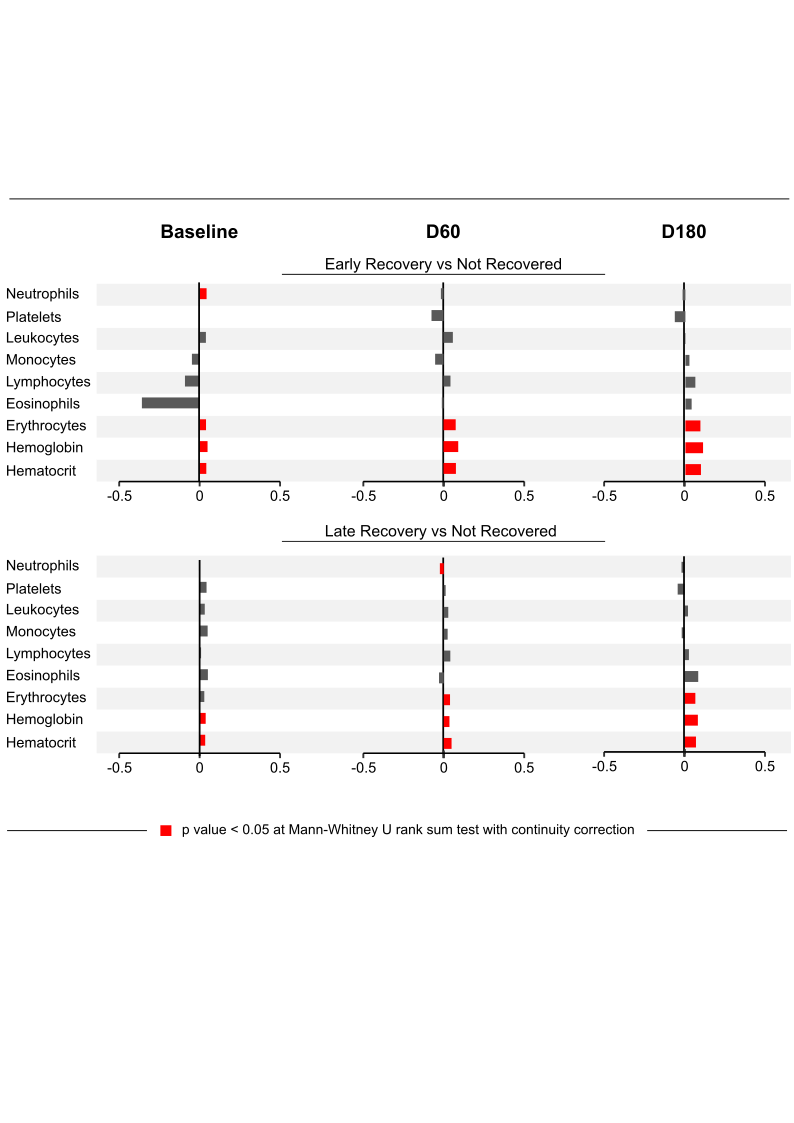


**Supplementary Figure 1. Differential change in complete cell blood counts between anemic and non-anemic patients.** Patients that were anemic at baseline (n=161) were divided according to recovery in Not Recovered, Early Recovery (D60), Late Recovery (D180) and Anemic at Baseline (were not anemic only at D60). A log_10_ of fold-change was calculated and statistical analyses were performed using the Mann-Whitney *U* adjusted test. Significative differences (p < 0.05) between groups for each time point are highlighted in red bars. Data are from 191 patients who had complete information on cell counts and biochemical measurements at all study timepoints.


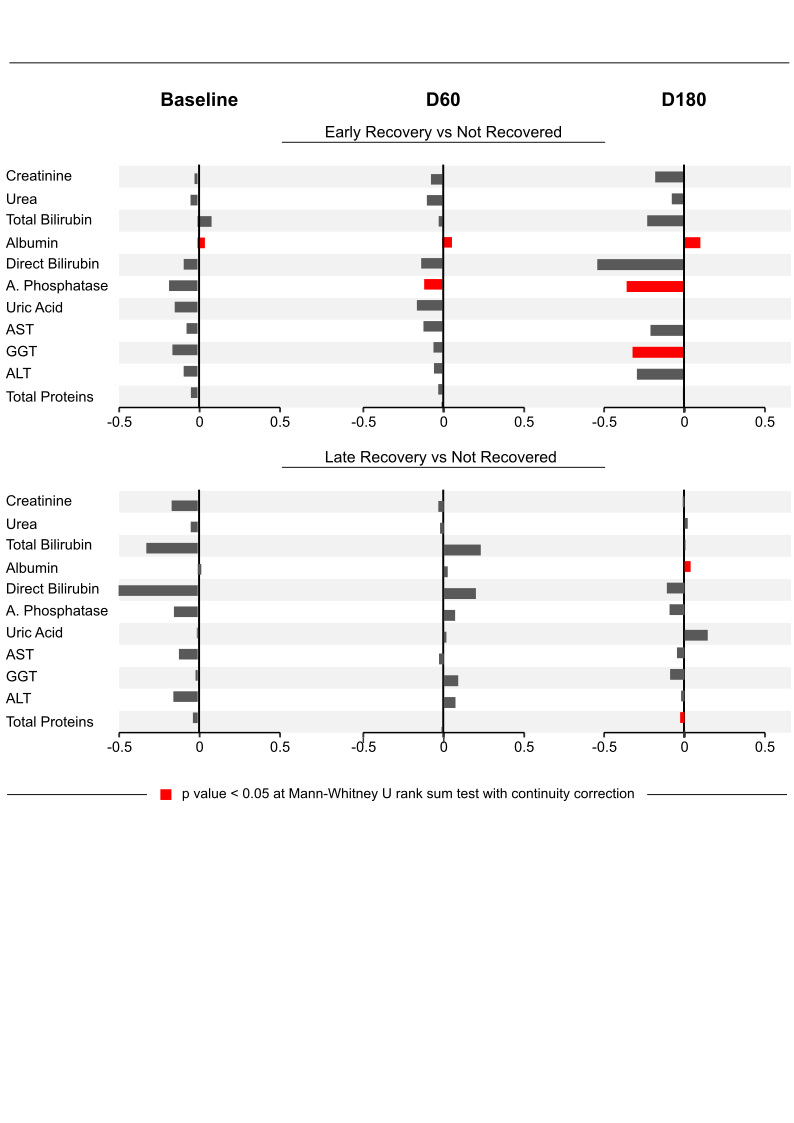


**Supplementary Figure 2. Differential change in biochemical markers between subgroups of recovery in patients that were anemic at baseline.** Patients that were anemic at baseline (n=161) were divided according to recovery in Not Recovered, Early Recovery (D60), Late Recovery (D180) and Anemic at Baseline (were not anemic only at D60). A log_10_ of fold-change was calculated and statistical analyses were performed using the Mann-Whitney *U* adjusted test. Significative differences (p < 0.05) between groups for each time point are highlighted in red bars. Data are from 191 patients who had complete information on cell counts and biochemical measurements at all study timepoints.
